# Why only 3 months? Immunological constraints on CAR-T therapy in gastrointestinal cancers

**DOI:** 10.3389/fimmu.2026.1942381

**Published:** 2026-10-05

**Authors:** Liping Zeng, Sheng Wei, Nan Chen, Xiangping Xie

**Affiliations:** 1 Department of Infectious Disease, Shaoyang Central Hospital, Shaoyang, China; 2 Department of General Medicine, the Second Affiliated Hospital of Wannan Medical College, Wuhu, China; 3 Department of Dermatology, Xuancheng People’s Hospital, Xuancheng, China

**Keywords:** CAR-T cell therapy, Claudin18.2, gastrointestinal tumours, immunotherapy for solid tumours, T-cell exhaustion, therapeutic window, tumour microenvironment

## Abstract

In June 2026, satri-cel/CT041, a chimeric antigen receptor (CAR) T-cell product targeting Claudin18.2, received approval from China’s National Medical Products Administration for the treatment of Claudin18.2-positive, HER2-negative advanced gastric/gastroesophageal junction adenocarcinoma after failure of at least two prior lines of therapy. This marks the first CAR-T product approved worldwide for solid tumours. The approval ends the long-standing debate over whether CAR-T cells can be effective against solid malignancies, yet it simultaneously raises a more pointed issue: in its pivotal randomised controlled trial, median progression-free survival was 3.25 months (95% CI 2.86-4.53) with satri-cel against 1.77 months (95% CI 1.61-2.04) with treatment of physician’s choice, giving a hazard ratio of 0.37 (95% CI 0.24-0.56). The two medians differ by 1.48 months; that figure marks where each survival distribution is cut in half and is not the benefit any individual patient can expect, and approval does not equate to a cure. Taking this as a starting point, this review systematically dissects the immunological bottlenecks that constrain CAR-T efficacy in gastrointestinal tumours. We first trace the evidence chain from phase I studies through the randomised controlled trial to exploratory adjuvant-therapy settings and then integrate the landscape of targets—Claudin18.2, glypican-3, carcinoembryonic antigen, mesothelin, and CDH17—within a target-tumour matrix encompassing gastric cancer, colorectal cancer, hepatocellular carcinoma, and pancreatic cancer. The core section focuses on four intertwined immunological constraints: spatiotemporal heterogeneity and escape at the antigen level; a therapeutic window shaped jointly by epitope masking within tight junctions and immune privilege of the gastric mucosal stem cell niche—this mechanism not only explains why Claudin18.2-directed CAR-T cells exhibit an acceptable safety profile despite target expression on normal tissues but also provides a paradigm for rational selection of next-generation targets; a suppressive microenvironment composed of regulatory T cells, myeloid-derived suppressor cells, tumour-associated macrophages, transforming growth factor-β, adenosine, and a hypoxic and acidotic metabolic milieu; and CAR-T exhaustion driven by chronic antigen stimulation. We particularly emphasise that metabolic stress and exhaustion are not two parallel obstacles. We put forward a mechanistic model in which persistent antigen stimulation combined with intratumoral hypoxia acts as a “double hit”: through mitochondrial dysfunction and reactive oxygen species accumulation, the two inputs raise nuclear factor of activated T-cell (NFAT) activity and so drive the TOX/NR4A-dominated exhaustion program. This model is assembled from chronic-infection and mouse tumour systems and has not been tested in human CAR-T cells or in satri-cel-treated patients. We advance it as a framework for organising the four bottlenecks and for explaining why single-point interventions struggle to lift the efficacy ceiling, not as a demonstrated account. On this basis, we discuss strategies such as armouring, logic gating, transcription factor engineering, gene editing, and chemokine receptor modification, along with combination regimens involving checkpoint inhibition, oncolytic viruses, locoregional administration, and microbiome modulation, and we evaluate alternative platforms including CAR-macrophages, CAR-NK cells, and *in vivo* CAR-T generation. The watershed has been crossed, but the work of turning 3 months into 30 has only just begun.

## 1 Introduction

The digestive system is one of the most frequent sites of malignant neoplasms. Colorectal cancer, gastric cancer, liver cancer, and oesophageal cancer consistently rank among the leading causes of cancer incidence and mortality worldwide, whereas pancreatic cancer, despite a relatively lower incidence, accounts for a disproportionately large share of cancer-related deaths due to its extremely poor prognosis (1). In China, the disease spectrum is particularly heavy, shaped by factors such as *Helicobacter pylori* infection, chronic hepatitis B endemicity, and dietary patterns, with new cases and deaths from gastric, liver, and oesophageal cancers representing a considerable proportion of global totals (1, 2).

The therapeutic ceiling is equally clear. Curative-intent resection combined with perioperative chemotherapy remains the standard for resectable cases, yet recurrence rates remain high. In the advanced setting, chemotherapy regimens built on platinum, fluoropyrimidine, and taxane backbones, along with targeted therapies against HER2, VEGFR, and Claudin18.2, can prolong survival, but median overall survival is often still measured in months (3). Immune checkpoint inhibitors have altered the landscape for certain subsets—patients with deficient mismatch repair/microsatellite instability-high (dMMR/MSI-H) colorectal cancer, PD-L1-high gastric cancer, and a subset of hepatocellular carcinoma can achieve durable responses (4, 5). Specifically, in dMMR/MSI-H metastatic colorectal cancer, first-line pembrolizumab extended progression-free survival from 8.2 to 16.5 months (KEYNOTE-177) (6); in HER2-negative advanced gastric/gastroesophageal junction adenocarcinoma, nivolumab plus chemotherapy as first-line treatment improved overall survival (CheckMate-649) (7); and in unresectable hepatocellular carcinoma, atezolizumab plus bevacizumab outperformed sorafenib, reshaping the first-line paradigm (IMbrave150) (8). Nevertheless, the majority of microsatellite-stable (MSS) colorectal cancers and immunologically “cold” pancreatic cancers exhibit little to no response. This gap is precisely what adoptive cell therapy seeks to fill.

Since the concept of chimeric antigen receptors was first introduced (9), the success of CAR-T in B-cell haematological malignancies has been firmly established by a series of studies—from early demonstration of durable memory and potent antitumour activity in patients (10), to sustained remissions in acute leukaemia (11), and even decade-long remissions in chronic lymphocytic leukaemia (12), as well as multiple pivotal trials (13–15, 126). Yet, the extension to solid tumours long remained stalled, to the point that the question “are CAR-T cells fundamentally unsuitable for solid tumours?” became a genuine debate within the field. Between 2024 and 2026, this question was effectively put to rest by a coherent chain of evidence: satri-cel, targeting Claudin18.2, first reported its final phase I results in *Nature Medicine* (16) and then demonstrated a survival benefit through the world’s first randomised controlled trial of a CAR-T product for solid tumours (CT041-ST-01) (17), and ultimately received approval from China’s National Medical Products Administration on 22 June 2026, for the indication of Claudin18.2-positive, HER2-negative advanced gastric/gastroesophageal junction adenocarcinoma after failure of at least two prior lines of therapy—the first approved CAR-T product for solid tumours globally (18).

A closer scrutiny of the data from this milestone, however, reveals a far more sobering picture. In CT041-ST-01, the satri-cel group achieved a median progression-free survival of 3.25 months (95% CI 2.86-4.53), compared with 1.77 months (95% CI 1.61-2.04) in the control (investigator’s choice of therapy) group; the hazard ratio was 0.37 (95% CI 0.24-0.56; one-sided log-rank p<0.0001), and median overall survival was 7.92 months (95% CI 5.78-10.02) versus 5.49 months (95% CI 3.94-6.93; hazard ratio 0.69, 95% CI 0.46-1.05) (17). Two points about these figures should be kept in view throughout what follows. First, the 1.48-month gap between the medians compares two distributions at their midpoints; it is not the gain an individual patient receives, and the hazard ratio, which uses the whole of follow-up, is the better summary of the treatment effect. Second, the 3 months of our title refers to the median progression-free survival reached on satri-cel, not to the between-arm difference. The effect is statistically robust and clinically meaningful—a genuine benefit for a patient population that has failed at least two prior lines of therapy and in which nearly 70% present with peritoneal metastasis. Yet, when placed alongside the durable remissions, often lasting years, achieved by CD19-directed CAR-T in acute lymphoblastic leukaemia, the contrast is stark.

The central question in the field has therefore shifted. It is no longer “can CAR-T work in solid tumours?”—that question has been answered. Rather, it is: given that the target is correctly chosen and has obtained regulatory endorsement, what exactly constrains the efficacy to a magnitude of 3 months? And how can that 3 months be extended to 30? This is an immunological question, not a clinical trial design issue. Taking this as its main thread, this review provides a paradigm for the rational selection of next-generation targets. We note that satri-cel-anchored reviews of CAR-T in solid tumours already exist (130); the contribution intended here is deliberately narrower—a gastrointestinal focus, the therapeutic-window paradigm distilled from Claudin18.2, and a synthesis that places metabolic stress and exhaustion on a single NFAT-convergent causal chain rather than treating them as parallel obstacles.

## 2 Evidence chain: from feasibility to approval

### 2.1 Phase I: feasibility and safety boundaries

The phase I study of satri-cel (CT041-CG4006, NCT03874897) enrolled patients with Claudin18.2-positive advanced gastrointestinal tumours (**Figure 1**). The interim analysis had already suggested a favourable safety profile and preliminary efficacy (objective response rate 48.6%) (19), and the final results further confirmed that, among the 98 infused patients, the objective response rate was 38.8%, the disease control rate 91.8%, median progression-free survival 4.4 months, and median overall survival 8.8 months. In terms of safety, no dose-limiting toxicities, treatment-related deaths, or immune effector cell-associated neurotoxicity syndrome (ICANS) were observed; cytokine release syndrome (CRS) occurred in 96.9% of patients, all events grade 1-2; gastric mucosal injury occurred in 8.2% of patients (16).

**Figure 1:**
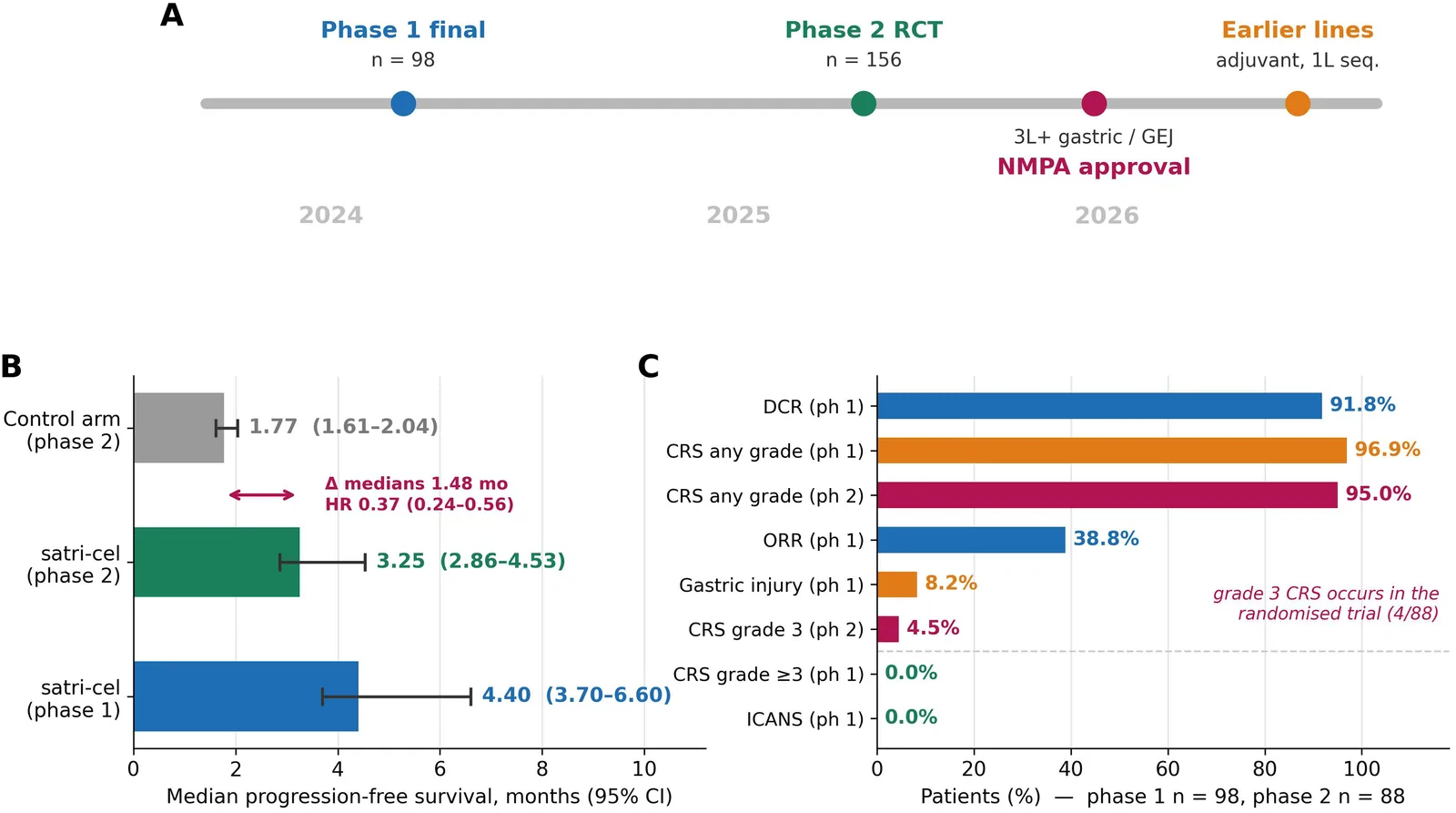
From proof of concept to the first approved solid-tumour CAR-T globally—and the persisting gap. **(A)** Milestones of the satri-cel program. **(B)** Median progression-free survival with 95% confidence intervals; the phase 1 and phase 2 cohorts are not directly comparable, and the 1.48-month gap between the two phase 2 medians summarises two distributions rather than an individual patient’s gain. **(C)** Phase 1 efficacy and safety (n = 98), with the phase 2 CRS grade distribution (n = 88) shown for contrast: grade 3 CRS occurred in the randomised trial but not in phase 1. CRS, cytokine release syndrome; ICANS, immune effector cell-associated neurotoxicity syndrome; ORR, objective response rate; DCR, disease control rate. Data from refs 16-18, 21, 22.

Two details in this dataset merit particular attention, as they foreshadow the immunological discussion to be developed later. First, CRS was common yet none reached grade ≥3—suggesting that CAR-T cells are effectively activated *in vivo*, but the intensity and duration of activation are subject to some constraint. Second, although the target antigen is expressed on normal gastric mucosa, the incidence of gastric mucosal injury was only 8.2% and manageable—a stark contrast to the fatal off-target toxicity previously caused by HER2-directed CAR-T (20). The mechanism underlying this difference is not a matter of chance but an inevitability rooted in the biology of the target (see Section 4.2).

### 2.2 Randomised controlled trial: confirmation of survival benefit

CT041-ST-01 (NCT04581473) is the first randomised controlled trial of a CAR-T product for solid tumours globally, conducted across 24 centres in China. Eligibility required Claudin18.2 immunohistochemistry intensity ≥2+ with ≥40% positive tumour cells, and failure of at least two prior lines of therapy. Between March 2022 and July 2024, 266 patients were screened, and 156 were randomly assigned in a 2:1 ratio to the satri-cel group (104 patients) or the investigator’s choice of therapy (TPC) group (52 patients); 88 and 48 patients, respectively, actually received treatment (17).

The refractory nature of the enrolled population is worth emphasising: approximately 69% of patients in the satri-cel group had peritoneal metastasis, and 27% had received three or more prior lines of therapy. Results showed that satri-cel prolonged progression-free survival (median 3.25 months, 95% CI 2.86-4.53, vs. 1.77 months, 95% CI 1.61-2.04; hazard ratio 0.37, 95% CI 0.24-0.56), meeting the primary endpoint; median overall survival was 7.92 months (95% CI 5.78-10.02) versus 5.49 months (95% CI 3.94-6.93), hazard ratio 0.69 (95% CI 0.46-1.05). Regarding safety, grade ≥3 treatment-emergent adverse events occurred in 99% of the satri-cel group versus 63% in the control group, predominantly comprising decreases in lymphocyte, white blood cell, and neutrophil counts. CRS was reported in 84 (95%) of 88 infused patients: grades 1–2 in 80 patients and grade 3 in four (4.5%), with no grade 4 or 5 event; one treatment-related death, from disseminated intravascular coagulation, occurred in the satri-cel arm (17). The grade distribution matters for the argument in Section 4.6. CRS after satri-cel is near-universal and usually mild, but it is not uniformly mild, and the phase I finding that every event fell within grade 1–2 does not carry over to the randomised setting.

### 2.3 Approval and advancement to earlier lines of therapy

On the basis of the above evidence, satri-cel received NMPA approval in June 2026, becoming the first approved CAR-T product for solid tumours globally (18). Concurrently, its application is being moved forward along two directions:

Adjuvant therapy in pancreatic cancer (CT041-ST-05, NCT05911217)—This is the world’s first study exploring CAR-T in the adjuvant setting for solid tumours. This phase Ib registrational trial enrols patients with Claudin18.2-positive, high-risk pancreatic ductal adenocarcinoma who have undergone curative-intent resection and adjuvant chemotherapy but present with abnormal CA19–9 levels without radiographic evidence of recurrence. Preliminary results were reported as a poster at the 2025 European Society for Medical Oncology (ESMO) congress: at a median follow-up of 6.05 months, only one of the six infused patients had relapsed, and median disease-free survival and overall survival had not been reached (21). The sample size is small and follow-up short, warranting cautious interpretation; nevertheless, the conceptual significance lies in shifting the application of CAR-T from “clearing measurable lesions” to “clearing minimal residual disease”—precisely the scenario in which CAR-T excels in haematological malignancies.

Perioperative and post-first-line sequential therapy in gastric cancer—A number of investigator-initiated trials are evaluating satri-cel as consolidation therapy after curative-intent resection (CT041-CG4010, NCT06857786) and as sequential therapy after first-line treatment (CT041-CG4011, NCT07179484) (18). In addition, the North American phase Ib/II study (CT041-ST-02, NCT04404595) is progressing with international population data, and preliminary results from its salvage cohort have been reported at the American Society of Clinical Oncology annual meeting (22).

The rationale for moving CAR-T to earlier lines is fundamentally immunological: at lower disease burden, antigen escape has not been fully selected for, the patient’s T-cell reserve and immune status are better, and the degree of microenvironmental suppression is relatively less severe. More importantly, as Section 4.4 argues, lower antigen load translates into weaker persistent stimulation—and persistent stimulation is the upstream driver of the exhaustion program. In other words, using CAR-T earlier not only exposes it to fewer tumour cells but also places it in a signalling environment that has not yet driven it into exhaustion.

## 3 Target-tumour matrix

Target selection largely determines the success or failure of CAR-T therapy in solid tumours. In this section, we synthesise the current landscape within a target-tumour matrix (summarised in **Tables 1** and **2**), with targets as one dimension and tumour types as the other.

**Table 1:** Representative clinical trials of the satri-cel (CLDN18.2 CAR-T) program in gastrointestinal cancers; NCT identifiers as reported in the cited primary sources.

Trial (NCT)	Product · target	Phase · setting	Population	Key efficacy/status	Ref
CT041-CG4006 (NCT03874897)	satri-cel · CLDN18.2	Phase 1, final	CLDN18.2+ advanced GI cancers	ORR 38.8%, DCR 91.8%, mPFS 4.4 mo, mOS 8.8 mo	(16, 19)
CT041-ST-01 (NCT04581473)	satri-cel · CLDN18.2	Phase 2 RCT, ≥2 prior lines	CLDN18.2+ HER2− gastric/GEJ adenocarcinoma	mPFS 3.25 mo (95% CI 2.86-4.53) vs. 1.77 mo (95% CI 1.61-2.04); HR 0.37 (95% CI 0.24-0.56); grade 3 CRS 4/88	(17)
CT041-ST-05 (NCT05911217)	satri-cel · CLDN18.2	Phase 1b, adjuvant	High-risk resected PDAC, CA19-9+, no radiographic recurrence	1/6 relapsed at 6.05 mo (ESMO 2025 poster)	(21)
CT041-ST-02 (NCT04404595)	satri-cel · CLDN18.2	Phase 1b/2, salvage (N. America)	Advanced gastric/pancreatic adenocarcinoma	Salvage cohort reported (ASCO)	(22)
CT041-CG4010 (NCT06857786)	satri-cel · CLDN18.2	IIT, post-resection consolidation	Gastric, curative-intent resection	Ongoing	(18)
CT041-CG4011 (NCT07179484)	satri-cel · CLDN18.2	IIT, post-first-line sequential	Gastric	Ongoing	(18)

Earlier-phase programs against other targets (GPC3, CEA/GUCY2C, mesothelin, CDH17, B7-H3) are discussed with primary citations in Section 3. GEJ, gastro-oesophageal junction; PDAC, pancreatic ductal adenocarcinoma; IIT, investigator-initiated trial; ORR, objective response rate; DCR, disease control rate; mPFS/mOS, median progression-free/overall survival.

**Table 2:** Expression characteristics of major CAR-T targets in gastrointestinal tumours, three assessments of the therapeutic window, and representative products.

Target	Normal-tissue expression	Expression in GI tumours	Therapeutic window (masked/stem-spared/regenerable)	Representative CAR-T products	Ref
CLDN18.2	Differentiated gastric mucosa only; epitope buried in tight junctions; stem-cell zone negative	Gastric, GEJ, pancreatic, oesophageal (epitope exposed on loss of polarity)	✓/✓/✓ WIDE — self-limiting gastric mucosal injury (8.2%, phase I)	satri-cel/CT041 (approved); VHH-based CARs	(16, 23, 24)
GPC3	Foetal liver; silenced postnatally	~70% of hepatocellular carcinoma	–/–/– WIDE — oncofoetal reexpression	GPC3 CAR-T; IL-15-armoured; AZD5851 (TGFβRII-DN)	(27–29)
CEA (CEACAM5)	Basal, accessible expression in normal colonic epithelium	Colorectal and other GI adenocarcinomas	✗/~/✓ NARROW — dose-dependent colitis	CEA CAR-T (regional delivery to limit systemic exposure)	(30, 31)
MSLN	Low in normal mesothelium	Pancreatic, cholangiocarcinoma	~/–/~ MODERATE	MSLN CAR-T	(33, 34)
HER2 (ERBB2)	Low-level, accessible on normal epithelia including lung	Subset of gastric/oesophageal	✗/✗/✗ NARROW — fatal off-tumour toxicity reported with high-affinity CAR	HER2 CAR-T (affinity tuning/logic gating required)	(20)
CDH17	Intestinal epithelium	Colorectal, gastric, neuroendocrine	~/–/✓ Under evaluation	CDH17 CAR-T	(36)
B7-H3 (CD276)	Restricted in normal tissue	Broad solid tumours incl. GI and tumour vasculature	–/–/– Promising, needs validation	B7-H3 CAR-T	(37, 38)
EpCAM/MUC1	Broad, accessible epithelial expression	Broad GI expression	✗/✗/~ NARROW	Limited progress; logic gating or local delivery needed	(105, 130)

Therapeutic-window criteria (see Section 4.2): (i) is the epitope structurally masked in normal tissue? (ii) is the stem/progenitor pool spared? (iii) is the tissue regenerable and the injury monitorable? ✓, criterion met; ✗, not met; ~, partial; –, not applicable (antigen essentially absent from normal tissue). CLDN18.2 satisfies all three, which is why a CAR-T against a normally expressed antigen produces only self-limiting mucosal injury. GEJ, gastro-oesophageal junction; VHH, single-domain antibody; DN, dominant-negative. Primary references for each target are listed in the final column. The two window types are not mechanistically equivalent: CLDN18.2 and CDH17 are epitope-masked targets, where the window rests on conformational inaccessibility in polarised epithelium, whereas GPC3 is an oncofoetal target, where it rests on postnatal transcriptional silencing (Section 4.2). Criterion (i) is decisive for the former and not applicable to the latter.

### 3.1 Claudin18.2: gastric and pancreatic cancer

Claudin18.2 is a splice isoform of the tight-junction protein Claudin-18. Its normal expression is strictly confined to differentiated epithelial cells of the gastric mucosa and is absent from the gastric mucosal stem cell zone. During malignant transformation, loss of cell polarity exposes epitopes that are otherwise buried within tight junctions, whereas expression may also ectopically extend to tumours of the pancreas, oesophagus, ovary, and lung (23). This property of “restricted in normal tissues, exposed in tumours” renders it a rare target in gastrointestinal malignancies that possesses a viable therapeutic window and underpins its parallel development across multiple modalities (monoclonal antibody zolbetuximab, bispecific antibodies, antibody–drug conjugates, and CAR-T).

In the CAR-T field, preclinical foundational studies on Claudin18.2-directed CAR-T confirmed, across 24 normal human tissues, that its expression is limited to gastric mucosa, providing the initial basis for the safety profile of this target (24); on this foundation, satri-cel became the only product to have completed the full development path (see Section 2). Of note are the differences in immunohistochemical cutoff values across modalities: CAR-T (CT041-ST-01) adopted a threshold of ≥2+ intensity with ≥40% positive cells (17), whereas the clinical cutoff for zolbetuximab is ≥2+ intensity with ≥75% positive cells—this monoclonal antibody combined with chemotherapy has established first-line survival benefit in two phase III trials (SPOTLIGHT and GLOW) (25, 26). These differing cutoffs mean that “Claudin18.2-positive” designates different populations in different contexts—this is not a mere technical detail but a practical issue requiring standardisation of companion diagnostics, and it directly affects cross-trial comparisons of efficacy. In addition, next-generation Claudin18.2-directed CAR-T constructs incorporating VHH (single-domain antibody) binding domains are currently under clinical evaluation.

### 3.2 GPC3: hepatocellular carcinoma

Glypican-3 (GPC3) is a membrane-anchored heparan sulphate proteoglycan that is expressed in foetal liver, silenced after birth, and reactivated in approximately 70% of hepatocellular carcinomas, while being virtually absent from normal liver (27). This oncofoetal pattern provides a favourable rationale for target selection. Early clinical studies confirmed the feasibility and preliminary activity of GPC3-directed CAR-T, but responses were limited by insufficient persistence and microenvironmental suppression (28). Targeted modifications include co-expression of interleukin-15 to support survival and memory differentiation—IL-15-armoured GPC3 CAR-T has undergone clinical validation in patients with solid tumours (29); incorporation of a transforming growth factor-β dominant-negative receptor (TGFβRII-DN) to counteract the TGF-β-rich suppressive microenvironment of hepatocellular carcinoma; and dual-targeting strategies against both GPC3 and AXL to address heterogeneity.

### 3.3 CEA and GUCY2C: colorectal cancer

Carcinoembryonic antigen (CEA/CEACAM5) is highly expressed in colorectal cancer, but normal intestinal epithelium also exhibits basal expression, posing a real risk of “on-target, off-tumour” toxicity; early systemic infusion studies observed that anti-CEA CAR-T could mediate regression of metastatic colorectal cancer but concurrently induced severe, transient colitis (30, 31). Accordingly, for the predominant metastatic patterns in colorectal cancer, locoregional administration has become the main direction: intrahepatic arterial infusion for liver metastases and intraperitoneal delivery for peritoneal metastases, which increase local cell concentrations while reducing systemic exposure; subsequent studies have further employed pressure-enhanced delivery technologies to improve intratumoural delivery efficiency (32). Guanylate cyclase C (GUCY2C) is another candidate target, and GCC19CART directed against it has completed phase I dose escalation in the United States.

A critical stratification factor in colorectal cancer is microsatellite status: dMMR/MSI-H tumours exhibit active immune infiltration and are theoretically more favourable for CAR-T efficacy, whereas the majority of MSS tumours display a “cold” phenotype, in which CAR-T monotherapy is unlikely to be effective and combinatorial strategies are required to actively “heat” the microenvironment (see Section 5.4).

### 3.4 Other targets

Mesothelin (MSLN) is highly expressed in pancreatic ductal adenocarcinoma and cholangiocarcinoma, with a low expression on normal mesothelium, making it an important candidate for CAR-T in pancreatic cancer; mRNA-based transiently expressed CAR-T targeting mesothelin has completed early-phase clinical studies in pancreatic cancer and mesothelioma without observable off-target toxicity (33, 34); a dual-target strategy combining mesothelin and CD19 has also undergone early clinical exploration in metastatic pancreatic cancer (35). HER2 is overexpressed in subsets of gastric and oesophageal cancers, but a high-affinity HER2-directed CAR-T previously caused fatal off-target toxicity due to low-level expression on pulmonary epithelium (20), defining a safety boundary. CDH17 (cadherin-17) is a particularly noteworthy target: although it is also expressed in normal intestinal epithelium, its molecular localisation within tight junctions between normal epithelial cells renders it “sequestered” and inaccessible to CAR-T; thus, CDH17-directed CAR-T can eliminate neuroendocrine tumours and gastric, pancreatic, and colorectal cancers without damaging normal intestinal tissue (36). This “expressed but masked” pattern closely parallels that of Claudin18.2 (see Section 4.2), and CDH17-directed CAR-T has entered early clinical development. B7-H3 (CD276) is overexpressed in a broad range of solid tumours and their vasculature while being restricted in normal tissues; CAR-T targeting B7-H3 has demonstrated potent antitumour activity across multiple solid tumour models without overt toxicity, and its efficacy exhibits a clear antigen-density dependence—further illustrating the therapeutic window in the presence of low-level normal tissue expression (37, 38). EpCAM and MUC1 have seen limited progress owing to widespread co-expression on normal epithelium with accessible epitopes, resulting in a narrow therapeutic window.

### 3.5 General principles of target strategy

The above landscape reflects the fundamental tension inherent in CAR-T therapy for solid tumours: truly tumour-specific antigens (TSAs) are virtually non-existent, and the accessible targets are almost all tumour-associated antigens (TAAs) that are expressed to some degree in normal tissues. Target selection therefore amounts to solving for the balance between “therapeutic coverage” and “safety margin”. Claudin18.2 stands out not because it is absent from normal tissues, but because its expression is subject to three layers of constraints—spatial, structural, and tissue-renewal-related. This is precisely the “therapeutic window enigma” that the next section will unfold, and it represents the most instructive design principle to be distilled from the current field.

## 4 Why only 3 months: dissection of the immunological bottlenecks

This section constitutes the core of the review. The factors limiting CAR-T efficacy in gastrointestinal tumours can be classified into four dimensions: antigen, microenvironment, the intrinsic fate of CAR-T cells (exhaustion), and physical barriers (**Figure 2**). We argue, however, that these four are not independent parallel obstacles. Section 4.4 sets out a molecular causal chain that we propose links metabolic stress in the microenvironment to the exhaustion program; if the model holds, it accounts for the difficulty of breaking the efficacy ceiling with single-point interventions. The chain is a hypothesis assembled from work in other systems, and the limits of the supporting evidence are set out in Section 4.4.3.

**Figure 2:**
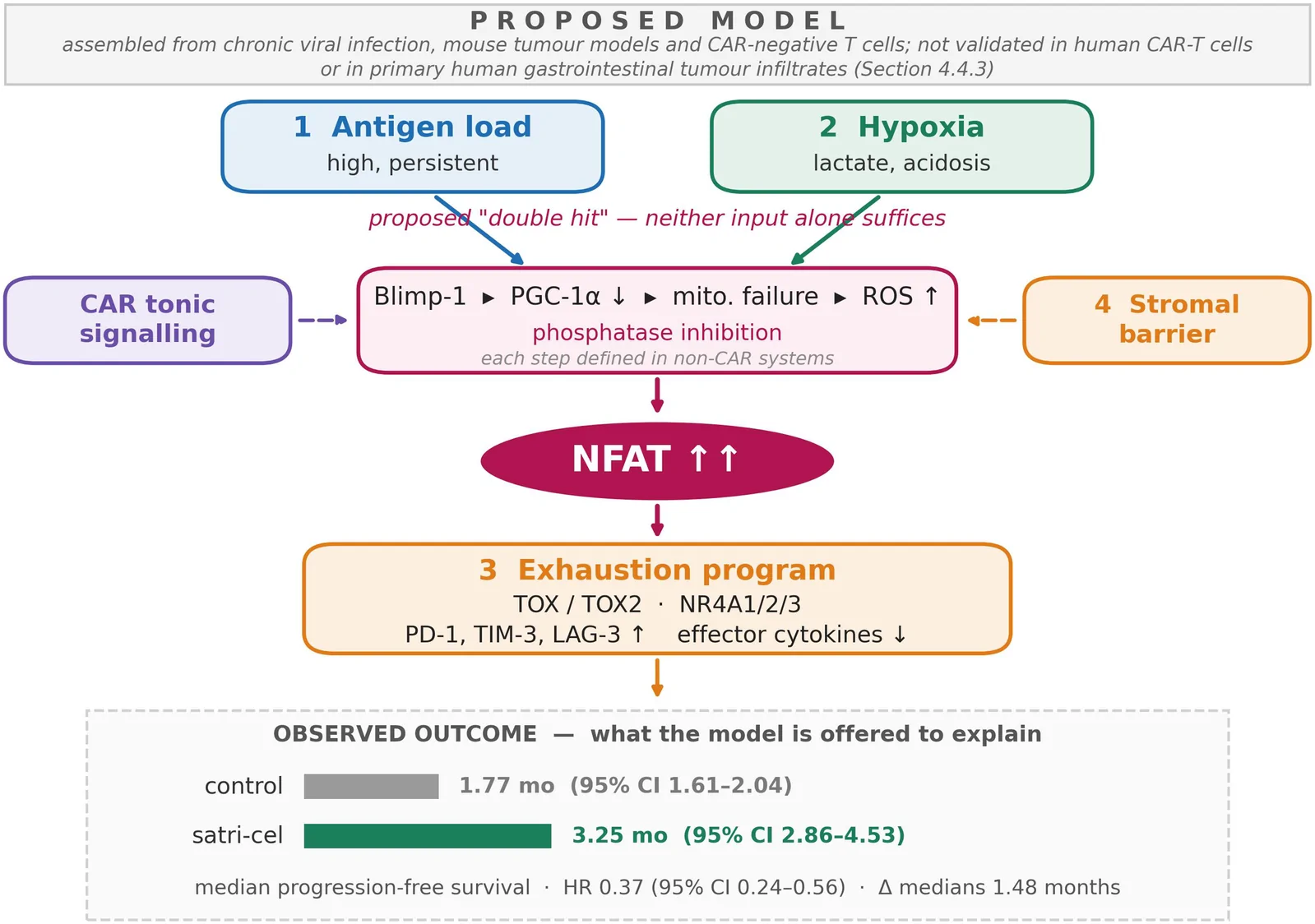
Proposed model: the four constraints converge on NFAT rather than acting in parallel. In the model advanced here, high antigen load and intratumoural hypoxia together drive the chain Blimp-1 → PGC-1α ↓ → mitochondrial failure → ROS ↑ → NFAT ↑↑, which activates the TOX/NR4A exhaustion program, with CAR tonic signalling and the stromal barrier feeding the same chain. The individual steps were defined in chronic viral infection, mouse tumour models, and T cells that do not carry a CAR; the sequence has not been validated in human CAR-T cells or in primary human gastrointestinal tumour infiltrates, and no metabolic or exhaustion data from satri-cel-treated patients are available (Section 4.4.3). The clinical figures at the foot of the panel—median progression-free survival 3.25 months (95% CI 2.86-4.53) versus 1.77 months (95% CI 1.61-2.04), hazard ratio 0.37 (95% CI 0.24-0.56)—are the outcome the model is offered to explain, not evidence that the chain operates.

### 4.1 The antigen dimension: heterogeneity, escape, and threshold

Spatial heterogeneity: Within the same tumour, target antigen abundance varies markedly across different regions and individual cells. CAR-T-mediated killing exhibits an antigen-density threshold effect—below a certain surface antigen copy number, the CAR fails to form an effective immunological synapse and sufficient signalling strength, allowing tumour cells to survive. Structural modifications of the CAR can lower this density threshold to a certain extent, and the choice of costimulatory domain also influences its responsiveness to low-antigen cells (39). This means that single-target CAR-T can, from the outset, only clear high-expressing subpopulations.

Escape over time: Under therapeutic pressure, antigens can undergo downregulation, splice variation, or complete loss. In haematological malignancies, CD19-negative relapse is one of the major mechanisms of CAR-T failure—its molecular basis includes acquired mutations and alternative splicing of CD19 (40), as well as broader mechanisms of target antigen loss (41). In solid tumours, a single infusion of EGFRvIII-directed CAR-T has also been observed to induce antigen loss and adaptive resistance (42), and CAR engagement itself can strip target antigen from the tumour surface by trogocytosis, lowering antigen density below the killing threshold while promoting fratricide and exhaustion among the CAR-T cells that acquired it (43). In solid tumours, where baseline heterogeneity is greater, the scope for antigen escape is correspondingly larger. The selective expansion of low- and negative-expressing clones is a plausible contributor to the short median progression-free survival achieved with satri-cel.

Cutoffs define populations: As noted in Section 3.1, the definition of Claudin18.2 positivity is not consistent across different modalities (17, 44). Using ≥40% versus ≥75% positive cells as the eligibility threshold not only alters the size of the treatable population but also changes the average antigen density of the enrolled cohort—and consequently the expected therapeutic efficacy. Standardisation of companion diagnostics is a prerequisite for cross-trial comparability and population optimisation.

### 4.2 The therapeutic window enigma: why Claudin18.2-directed CAR-T does not destroy the stomach

The observation looks paradoxical: Claudin18.2 is expressed on normal gastric mucosa, and CAR-T cells do not distinguish between “normal” and “tumour”—one would therefore expect severe gastric mucosal destruction. Yet, in the phase I study, gastric mucosal injury was observed in only 8.2% of patients and was manageable (16), in sharp contrast to the fatal pulmonary toxicity caused by HER2-directed CAR-T (20). Understanding this discrepancy is the key to understanding the entire logic of target design. The triple mechanism collectively defines this therapeutic window.

First is structural epitope masking. In intact normal gastric mucosa, Claudin18.2, as a component of tight junctions, has its extracellular loop buried within the junctional structures between adjacent cells, rendering it largely inaccessible to antibodies (and to the CAR’s scFv). During malignant transformation, however, epithelial polarity is lost and tight junctions disintegrate, exposing the epitope on the cell surface (23). In other words, this is a target defined by cellular polarity status rather than by expression level per se—the difference between normal and tumour tissues lies not in “presence or absence,” but in “accessibility”.

Second is sparing of the stem cell zone. Claudin18.2 is expressed on differentiated gastric mucosal epithelial cells, but not on gastric mucosal stem cells (23). This means that even if the differentiated epithelium is eliminated by CAR-T cells, the stem cell pool remains intact and the mucosa can regenerate. The resulting injury is therefore self-limiting and repairable—in stark contrast to the irreversible organ failure caused by HER2-directed CAR-T attacking pulmonary epithelium.

Third is tissue renewability and accessibility. The gastric mucosa is a high-turnover tissue, and its injury can be clinically monitored endoscopically and managed with supportive care. In contrast, organs such as lung and myocardium lack such regenerative capacity and do not offer equivalent monitoring or intervention options.

Together, these three points constitute a generalisable screening paradigm for target selection: when evaluating whether a TAA is druggable, one should not merely ask whether it is expressed in normal tissues, but rather pose three questions—(i) is the epitope structurally masked in normal tissues? (ii) is the stem/progenitor cell pool of that tissue spared? and (iii) is the tissue regenerable, and is its injury monitorable and manageable? Applying this standard retrospectively, HER2 fails on all three counts; CEA is expressed on normal intestinal epithelium with accessible epitopes (hence colitis (30)), whereas Claudin18.2 satisfies all three criteria. Importantly, Claudin18.2 is not an isolated case. CDH17 provides an independent validation: it is also expressed in normal intestinal epithelium, yet similarly sequestered within tight junctions and inaccessible to CAR-T cells; consequently, CDH17-directed CAR-T can eliminate gastrointestinal tumours in preclinical models without damaging normal intestinal tissue (36). Two targets that are unrelated in both chemical structure and biological function share the same safety logic of tight-junction masking—indicating that the above paradigm is not a *post-hoc* explanation for a single fortunate target, but rather a general principle applicable to prospective screening. This is particularly valuable for gastrointestinal tumours: tight junctions are a universal structure of epithelial tissues, and loss of polarity during malignant transformation is a common event in epithelial carcinogenesis; therefore, “polarity-dependent epitope exposure” likely represents a class of target-rich resources that have not yet been fully exploited. This framework has direct guiding value for the rational selection of next-generation targets (see **Table 2**).

A distinction that has so far been left implicit deserves to be made explicitly, because epitope-masked and oncofoetal targets create a therapeutic window by different means and therefore fail in different ways. For CLDN18.2 and CDH17, the antigen is genuinely present in normal epithelium and is transcribed there at levels comparable with those in tumour; what separates the two compartments is conformational accessibility, since the extracellular loop sits within an intact junctional complex and becomes visible to an scFv only once polarity is lost (23, 36). The window is thus structural and close to all-or-none, and it is contingent on junctional integrity: any condition that transiently opens tight junctions in normal mucosa—erosive gastritis, Helicobacter pylori infection, or the lymphodepleting regimen itself—should in principle unmask the epitope and widen the on-target field. That contingency is the most economical explanation for why gastric mucosal injury occurred in 8.2% of patients rather than in none at all (16). Affinity tuning offers little protection in this setting, because once the epitope is exposed, the antigen density on normal and malignant cells is broadly similar. For GPC3, the logic is different in kind. The protein is silenced after birth by developmental transcriptional and epigenetic programmes and is reexpressed in roughly 70% of hepatocellular carcinomas (27); on the adult hepatocyte, the epitope is not hidden but absent. This window is quantitative rather than structural, which makes it indifferent to inflammation and to loss of polarity, and it is precisely the setting in which affinity and antigen-density thresholds can be calibrated to spare tissue carrying low-level residual expression (39). Its characteristic failure mode differs accordingly: regenerating tissue that reactivates a foetal programme may reexpress the antigen, so risk tracks with tissue turnover state rather than with junctional damage. The practical corollary is that the three questions above do not carry equal weight for the two classes. Criterion (i) is decisive for masked targets and simply not applicable to oncofoetal ones, whereas criteria (ii) and (iii)—stem-cell sparing and monitorable regeneration—carry most of the weight for oncofoetal targets, whose margin of safety depends entirely on what happens if silencing is reversed. **Table 2** and **Figures 3A–C** are annotated on this basis.

**Figure 3:**
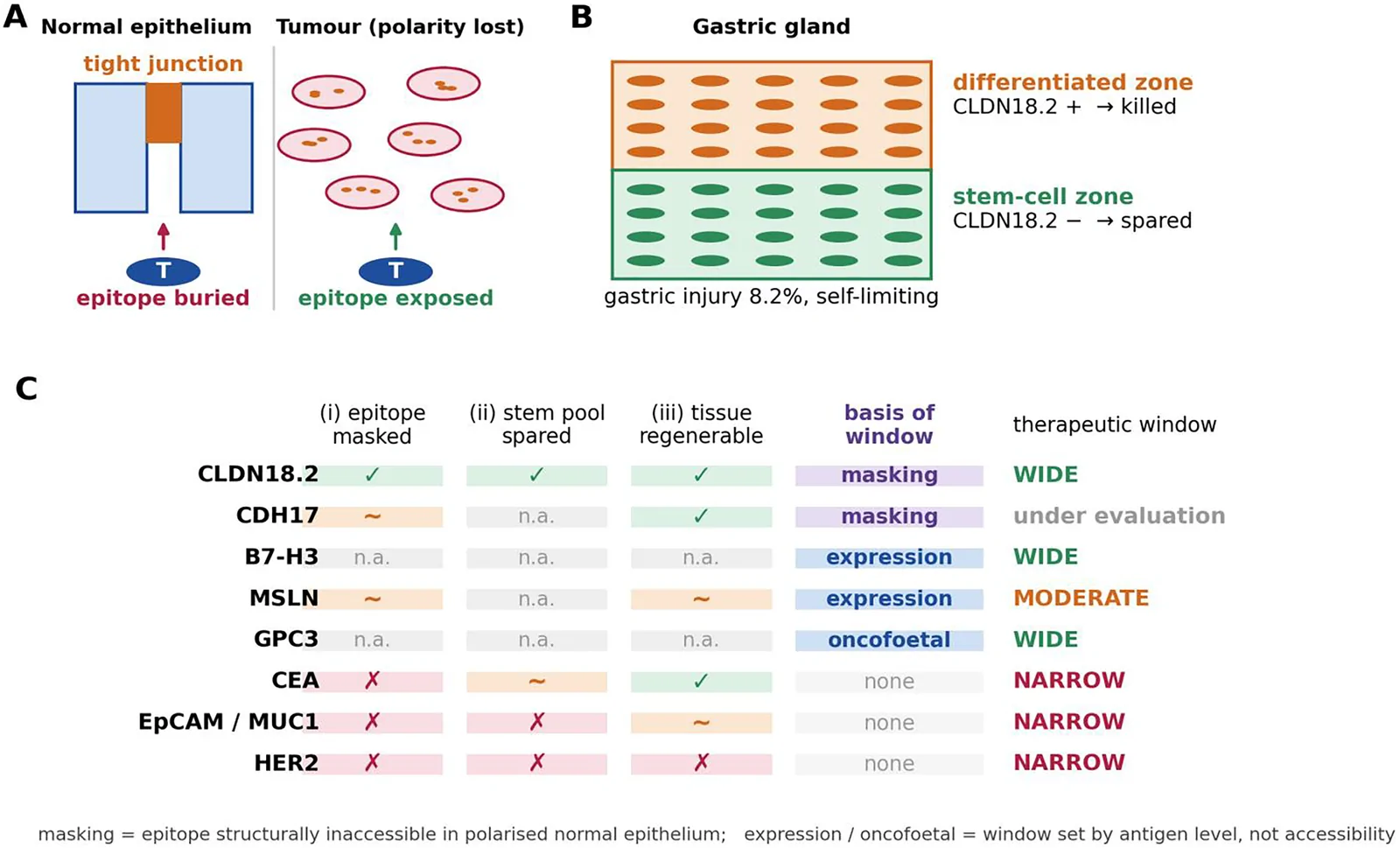
The enigma of the Claudin18.2 therapeutic window: why a CAR-T product targeting an antigen also expressed in normal tissues does not destroy the stomach. **(A)** Tight junctions bury the epitope in normal epithelium; loss of polarity in tumour exposes it. **(B)** CAR-T clears the CLDN18.2-positive differentiated zone but spares the CLDN18.2-negative stem-cell zone, so the mucosa regenerates. **(C)** Three-question screening paradigm applied to eight targets: (i) is the epitope structurally masked in normal tissue, (ii) is the stem or progenitor pool spared, (iii) is the tissue regenerable and the injury monitorable. ✓, met; ~, partial; ✗, not met; n.a., not applicable (antigen essentially absent from normal tissue). The fourth column records the basis of the window—structural masking of the epitope, quantitative separation of expression level, oncofoetal silencing, or none—since these are not mechanistically equivalent and fail under different conditions (Section 4.2).

### 4.3 The microenvironment: a suppressive network with a metabolic root

#### 4.3.1 Metabolism: an asymmetric hostility

Solid tumours are broadly hypoxic. Tumour cells glycolyse vigorously, which acidifies the milieu through lactate accumulation and at the same time deprives infiltrating T cells of glucose—this metabolic competition itself drives tumour progression (45, 46). T-cell effector function depends heavily on glycolysis, whereas memory differentiation relies on fatty acid oxidation and mitochondrial fitness. Glucose deprivation, lactate accumulation, and severe hypoxia therefore remodel T-cell energy metabolism. Effector cytokine secretion and proliferative capacity both decline as a result (47). Notably, elevated tumour glycolysis marks immune resistance to adoptive cell therapy (48)—a finding that links metabolic constraints directly to CAR-T efficacy rather than to the generic concept of “T-cell function”. Hypoxia also acts through a defined molecular pathway. Hypoxia-inducible factor HIF-1α directly upregulates PD-L1 on myeloid cells and tumour cells, which reinforces the suppressive activity of MDSCs (49)—a step that couples the metabolic layer to the cellular suppressive components discussed below.

The more important point is that metabolic stress is asymmetric. The very same metabolic milieu that impairs effector cells actively reinforces suppressive cells. (1) Lactate blunts the immune surveillance functions of T cells and NK cells (47). (2) Tumour-infiltrating regulatory T cells (Tregs), by contrast, use lactate to sustain their suppressive function—deleting the lactate transporter MCT1 in Tregs reduces tumour growth and synergises with checkpoint blockade (50). (3) Tumour-derived lactate also drives tumour-associated macrophages (TAMs) toward an M2-like polarisation (51).

The metabolic state of the microenvironment is therefore not passively unfavourable; it is a mechanism that converts metabolic disadvantage into immunosuppressive advantage. This asymmetry has an important practical implication: simply “replenishing energy” for CAR-T cells or enhancing their metabolic fitness is insufficient—because in the same environment, one is also fuelling Tregs and M2-polarised TAMs. Truly effective strategies must be selective (e.g., targeting lactate uptake in Tregs) or act at the source to reduce tumour glycolysis and acidification.

#### 4.3.2 Suppressive cells: the heterogeneity of Tregs cannot be overlooked

Regulatory T cells, myeloid-derived suppressor cells (MDSCs), and M2-polarised TAMs together constitute the cellular suppressive network of the microenvironment (52–54). In gastrointestinal tumours, however, a blanket description of Tregs can be misleading and warrants specific clarification.

In colorectal cancer, the relationship between FOXP3^+^ infiltration and prognosis has long been controversial—some studies have even reported an association with better prognosis. Recent work resolves this paradox: tumour-infiltrating FOXP3^+^CD4^+^ cells actually comprise two functionally opposing subsets—the suppressive FOXP3^hi^ Tregs (associated with poor prognosis) and the non-suppressive FOXP3^lo^ non-Treg cells that secrete inflammatory cytokines (associated with better prognosis) (55). Therefore, simply equating “FOXP3^+^ cell infiltration” with immunosuppression is not valid in colorectal cancer. Beyond this FOXP3^hi^/FOXP3^lo^ distinction, three further layers of Treg heterogeneity bear directly on gastrointestinal tumours yet are rarely separated in CAR-T discussions. First, tissue-resident Tregs adapted to the gut mucosa carry a distinct transcriptional and metabolic profile and are sustained by local cues, so systemically acting Treg-directed strategies may leave them untouched. Second, TIM-3^+^ (and other checkpoint-high) Treg subsets are typically the most strongly suppressive and effector-like and are enriched at the tumour site; they are also the fraction most readily co-engaged by checkpoint blockade, which can act on them in an unhelpful direction. Third, Treg plasticity—destabilisation toward inflammatory phenotypes under metabolic and cytokine stress—means the same microenvironmental levers noted above (lactate, adenosine, and TGF-β) act on Tregs and effector CAR-T cells at once and in opposite functional directions (54). Selectivity for the suppressive, tissue-resident, checkpoint-high fraction, rather than blanket Treg depletion, is therefore the more defensible engineering goal.

#### 4.3.3 Soluble factors: TGF-β and adenosine

TGF-β represents one of the most potent inhibitory axes in gastrointestinal tumours. It broadly suppresses T-cell proliferation and cytotoxic function and promotes Treg differentiation (56). A key study established its central role in gastrointestinal malignancies: mice carrying four common colorectal cancer mutations (Apc, Kras, Trp53, and Tgfbr2) recapitulate key features of human microsatellite-stable colorectal cancer—low mutational burden, T-cell exclusion, and TGF-β-activated stroma; in this model, PD-1/PD-L1 blockade elicited limited responses, whereas TGF-β inhibition unleashed robust and durable cytotoxic T-cell responses and prevented metastasis, and also rendered established liver metastases re-sensitive to checkpoint therapy (57). Human data likewise show that tumours non-responsive to PD-L1 blockade exhibit an “immune-excluded” phenotype (CD8^+^ T cells confined to the stroma rather than intratumoural), accompanied by upregulation of a fibroblast-derived TGF-β response gene signature (58).

This body of evidence elevates TGF-β from a generic inhibitory factor to a primary mechanism of immune evasion in gastrointestinal tumours (particularly MSS colorectal cancer)—which explains why incorporating a TGF-β dominant-negative receptor into CAR-T cells is not a universal manoeuvre, but a strategy with tumour-type-specific justification (see Section 5.3).

Adenosine axis: Extracellular ATP is converted to adenosine via the CD39/CD73 cascade, which signals through the A2A receptor (A2AR) to elevate intracellular cAMP and transmit a potent inhibitory signal (59). One study directly quantified the magnitude of its suppression on CAR-T cells—the degree of inhibition of human CAR-T interferon-γ production by an A2AR agonist is comparable with that of TGF-β (60). This suggests that adenosine is not a secondary factor but an obstacle on par with TGF-β and thus warrants equal attention in engineering strategies (see Section 5.3).

### 4.4 The intrinsic fate of CAR-T cells: the exhaustion program and its causal link to metabolism

Even when CAR-T cells successfully infiltrate and recognise antigen, they still face their own fate problem. Exhaustion is not simply “functional fatigue,” but rather a differentiation state with well-defined transcriptional, epigenetic, and metabolic features (61, 62).

#### 4.4.1 Transcriptional program: the NFAT-TOX/NR4A axis

The phenotypic hallmarks of exhaustion are sustained co-expression of inhibitory receptors (PD-1, TIM-3, LAG-3, and TIGIT)—among which high PD-1 expression and its blockade-mediated functional restoration were the earliest identified features of this state (63)—along with a graded loss of effector cytokine secretion capacity (61). The upstream cascade is now clearly defined: chronic T-cell receptor/CAR signalling, via the calcium-calcineurin-NFAT pathway, induces the high-mobility group proteins TOX and its homologue TOX2, as well as the NR4A family nuclear receptors (NR4A1/2/3). Once induced by NFAT2, TOX enters a calcineurin-independent feed-forward loop and persists in exhausted cells; loss of TOX reshapes this program and markedly attenuates the exhausted phenotype rather than abolishing exhaustion outright (64–68). TOX/TOX2 and NR4A act synergistically downstream of NFAT to impose this program (70).

Direct experimental evidence establishes the relevance of this axis to CAR-T efficacy in solid tumours. In CAR-T-bearing mouse tumour models, tumour-infiltrating CD8^+^CAR^+^PD-1^hi^TIM-3^hi^ cells highly expressed NR4A; triple NR4A-knockout CAR-T cells significantly reduced tumour burden and prolonged survival of tumour-bearing mice, with their transcriptional program shifted from exhaustion toward effector function (67), and independent studies have confirmed NR4A1 as a key mediator of T-cell dysfunction (72). TOX/TOX2 double-knockout CAR-engineered tumour-infiltrating lymphocytes similarly exhibited enhanced tumour-suppressive capacity and lower inhibitory receptor expression (70). This positions TOX/NR4A as the most mechanistically targeted intervention point for CAR-T engineering (see Section 5.3).

#### 4.4.2 Epigenetic dimension: an ongoing debate

Exhaustion is accompanied by large-scale remodelling of chromatin accessibility, forming an epigenetic landscape distinct from that of effector and memory T cells (74, 75). An influential body of work has argued that this landscape is “fixed”: PD-L1 blockade induces only transient transcriptional reprogramming, whereas the epigenetic imprint of exhaustion remains stable (69); tumour-specific T cells pass through two discrete chromatin states—a plastic dysfunctional state (which can be “rescued” by reprogramming) and a fixed dysfunctional state (resistant to reprogramming), distinguishable by surface markers (71), and *de novo* DNA methylation programs further impede PD-1 blockade-mediated “revival” (79).

This “irreversibility” view, however, has recently been challenged. In CAR-T cells, forced downregulation of CAR protein via a pharmacologically regulatable system, or reversible inhibition of CAR signalling with the multikinase inhibitor dasatinib—thereby inducing a transient pause (“rest”) in receptor signalling—even in CAR-T cells that had already acquired exhaustion features, just a few days of rest sufficed to induce a memory-like phenotype, global transcriptional and epigenetic remodelling, and restoration of antitumour function (73). The authors explicitly state that this challenges the view that exhaustion is an epigenetically fixed state.

How to reconcile these findings? We propose that the discrepancy may not lie in whether the epigenetic landscape is stable, but rather in whether signalling is continuous. In the experimental systems of Pauken and Philip et al., antigenic stimulation is persistent (chronic infection, progressing tumours), and checkpoint blockade relieves only inhibitory receptor signals, whereas the driving signal from the CAR/TCR itself is not interrupted; Weber et al., in contrast, directly interrupted the driving signal. In other words, the “fixation” of exhaustion is likely a state actively maintained by continuous signalling, rather than an irreversible end point once established. Exhausted subsets themselves exhibit a developmental hierarchy (progenitor → intermediate → terminal) (74), with reversibility decreasing along this hierarchy.

#### 4.4.3 The metabolic-exhaustion causal chain: why the four-dimensional bottlenecks reinforce each other

A central argument of this review is that metabolic stress (Section 4.3) and exhaustion are best treated not as two parallel bottlenecks but as points on one causal chain. We set this out as a proposed model; the evidence assembled below comes from systems other than human CAR-T cells, and the limits of the inference are stated at the end of this subsection. Within those systems, the findings are consistent: persistent antigen stimulation and hypoxia alone each allow CD8^+^ T cells to differentiate into functional effector cells; only when both are combined does dysfunction meeting the definition of exhaustion occur rapidly (76, 83). The proposed mechanism runs as follows: persistent stimulation, acting through Blimp-1, inhibits PGC-1α-dependent mitochondrial reprogramming, so that cells cannot adapt to hypoxia; loss of mitochondrial function allows reactive oxygen species (ROS) to accumulate to intolerable levels; and ROS raises NFAT activity by inhibiting phosphatases, feeding back into the NFAT-TOX/NR4A axis described in Section 4.4.1 (76). In addition, PD-1-induced bioenergetic defects are themselves early drivers of exhaustion rather than its consequence (85). We state plainly that this causal chain draws largely on chronic viral-infection models, mouse tumour models, and T-cell systems that do not carry a CAR; to our knowledge, the specific sequence Blimp-1 → PGC-1α↓ → ROS↑ → NFAT↑ has not been validated directly in human CAR-T cells or in primary human gastrointestinal tumour infiltrates, and species differences in T-cell metabolism and transcriptional control warrant caution. Human infusion-product studies in haematological CAR-T (132, 133) lend partial support to the transcriptional arm but do not address the metabolic arm, and no exhaustion or metabolic data from satri-cel-treated patients are yet available. The account offered here—that convergence on NFAT sets the 3-month efficacy ceiling—should therefore be read as the model we propose to organise the existing evidence, not as a mechanism demonstrated in the very product it is used to explain.

Two further limitations bear specifically on the step from these models to CAR-T cells in gastrointestinal tumours. The first is receptor biology. The chain set out above was defined for TCR-driven stimulation, and CAR signalling is not a synthetic copy of it. CARs engage MHC-independent ligands with nanomolar rather than micromolar affinity, recruit a compressed and partly non-canonical set of proximal intermediates, and generate a signal of different amplitude and duration from the TCR at equivalent antigen occupancy (138). The immunological synapse differs as well: instead of the concentric arrangement formed by the TCR, CARs assemble a disorganised multifocal Lck cluster that lacks a defined peripheral ring and recruits lytic granules more rapidly (139). A synapse that forms and dissolves on a different timescale implies a different duty cycle of calcium flux, and therefore of NFAT nuclear residence—the very variable on which the Blimp-1 to PGC-1alpha to ROS to NFAT chain converges. Whether chronic CAR stimulation delivers the same integrated NFAT dose as chronic TCR stimulation, over the same interval, has not been established. The second limitation is that the CAR contributes an input for which the TCR has no counterpart: antigen-independent tonic signalling arising from scFv self-association, which begins during ex vivo manufacture and is modulated by the costimulatory endodomain (Section 4.4.4) (77). Exhaustion kinetics in a CAR-T product therefore start from a non-zero baseline that is set before the cell ever encounters tumour, and the relative contributions of tonic signalling, tumour antigen load, and hypoxia to the final exhausted state have not been separated in any human gastrointestinal dataset. Neither point overturns the chain we propose, but together they mean that its timing—how much antigen exposure, over how many days, is needed to fix the program—cannot be carried across quantitatively from TCR-based models to satri-cel, and that a CAR-specific parameterisation will be required before the model can be used predictively.

If the model is correct, it would account for a phenomenon so far described only qualitatively: why CAR-T cells in solid tumours exhaust faster and more completely than in haematological malignancies. The proposed answer is not the tautology that “the solid tumour microenvironment is worse” but that solid tumours supply two conditions at once—high antigen load (persistent stimulation) and severe hypoxia (metabolic stress)—which on this account converge at the molecular level on NFAT and jointly ignite the exhaustion program. The implication that follows, and it stands or falls with the model, is that any intervention targeting a single dimension will be of limited efficacy. Improving infiltration alone, without alleviating hypoxia, will still lead to exhaustion once CAR-T cells arrive; knocking out PD-1 alone, without reducing antigen stimulation intensity or relieving metabolic stress, will still leave the NFAT-TOX axis driven.

#### 4.4.4 The CAR itself as a driver of exhaustion: tonic signalling

CAR-T cell exhaustion may begin even before infusion, a point that the field often overlooks. Antigen-independent aggregation of the scFv can trigger tonic phosphorylation of CD3ζ, delivering constitutive activation signals in the absence of antigen, thereby inducing early exhaustion during the ex vivo manufacturing phase and limiting post-infusion antitumour efficacy (77). Many of the CARs studied to date display this effect; certain CD19 constructs display comparatively low tonic signalling, which may contribute to their favourable persistence, although this is best regarded as a relative rather than an absolute distinction.

Critically, the choice of costimulatory domain modulates this process: CD28 costimulation exacerbates tonic-signalling-induced exhaustion, whereas 4-1BB costimulation mitigates it (77)—this provides a mechanistic explanation for the clinically observed superior persistence of 4-1BB-based CD19 CAR-T over CD28-based counterparts and directly informs CAR structural choices for solid tumours. More broadly, different costimulatory domains confer markedly different metabolic and differentiation fates upon CAR-T cells: CD28 drives vigorous glycolysis and rapid effector differentiation, whereas 4-1BB promotes fatty acid oxidation and memory maintenance (87, 88); other costimulatory molecules such as ICOS have also been explored to further optimise persistence (89).

Clinical studies have now validated two engineering strategies derived from these insights: (1) ITAM dose tuning: CD28/CD3ζ-based CARs contain three ITAMs, whose signalling redundancy can accelerate terminal differentiation and exhaustion. The 1XX design, which retains only the membrane-proximal functional ITAM (inactivating the two distal ones), achieves a balanced effector-memory program, preserving cytolytic capacity while mitigating exhaustion (78). (2) Targeted knock-in: Knocking the CAR construct into the TRAC locus enables physiological, antigen-inducible feedback-controlled CAR expression (rather than constitutive high expression from viral vectors), significantly reducing tonic signalling and delaying exhaustion (80).

Importantly, these are not preclinical concepts: the 1XX-designed CAR-T has completed a first-in-human phase I trial, achieving high response rates at low cell doses with a low incidence of severe CRS/ICANS (81)—demonstrating that signal tuning is a clinically validated pathway with realistic prospects for translation to solid tumours.

### 4.5 Physical barriers and insufficient infiltration

To exert their effects, CAR-T cells must first reach the tumour. The aberrant vasculature of solid tumours is structurally disorganised and poorly perfused and often downregulates the adhesion molecules and chemokine receptor ligands required for T-cell extravasation, making it difficult for infused cells to cross the vascular wall. Upon arrival, dense fibrotic stroma (desmoplasia) constitutes a second barrier—pancreatic ductal adenocarcinoma being the most extreme example, where stroma can account for the majority of tumour volume, and CAR-T cells, even when entering the tumour bed, are often “trapped” within the stroma and unable to contact tumour cells (93, 135). This physical constraint is a major reason for the prolonged stagnation of CAR-T in pancreatic cancer and explains why CAR-macrophages, which inherently possess tissue-infiltrating capacity, are regarded with high promise in this tumour type (see Section 5.5). Countermeasures are beginning to emerge: engineering Claudin18.2-directed CAR-T with the CXCR2 chemokine receptor significantly improved infiltration in pancreatic ductal adenocarcinoma and remodelled its immune microenvironment (94).

A frequently overlooked indirect effect of the stromal barrier is that it exacerbates intratumoural hypoxia and poor perfusion, thereby amplifying metabolic stress via the pathway described in Section 4.4.3. Physical barriers thus not only “block” cellular access but also provide fuel for exhaustion.

### 4.6 Immunobiology of toxicity

The combination of high incidence and generally low grade of CRS after satri-cel is worth interpreting, provided the two studies are kept apart. In the phase I study, all 95 patients with CRS among the 98 infused had grade 1–2 events and none reached grade 3; in the randomised trial CRS occurred in 84 (95%) of 88 infused patients, of whom four (4.5%) had grade 3 events, with none above grade 3 (16, 17). The driver of CRS is not primarily the CAR-T cells themselves, but rather the recruitment and activation of monocytes/macrophages by activated CAR-T cells, which in turn release large amounts of IL-6 and IL-1β, forming an amplification loop—this is the mechanistic basis for the effective control of CRS by the anti-IL-6 receptor monoclonal antibody tocilizumab. Previous work has systematically characterised the kinetics and biomarkers of CRS onset and has established a dedicated grading and management framework (95, 96). That CRS is near-universal yet predominantly low-grade, with grade 3 events confined to a small minority and none above grade 3, suggests that CAR-T cells are effectively activated but that the magnitude and duration of activation are constrained by tumour burden, antigen density, and the microenvironment—consistent with a median progression-free survival measured in months: the same set of constraints limits both toxicity and efficacy.

ICANS was not reported in the phase I study of satri-cel (16), contrasting with the relatively common neurotoxicity observed with CD19-directed CAR-T, likely related to the distribution of the target antigen and the scale of CAR-T expansion. From a practical standpoint, the management follows the mechanism. Because CRS is monocyte/macrophage-driven, grade ≥2 events are treated with the anti-IL-6-receptor antibody tocilizumab, with corticosteroids added for refractory or neurologic cases, along established grading and management frameworks (95, 96); the CRS seen with satri-cel, near-universal and grades 1–2 in the large majority but grade 3 in roughly one patient in 20 in the randomised trial, is generally controllable on this pathway. On-target gastric mucosal injury, being self-limiting and endoscopically monitorable (Section 4.2), is handled with proton-pump inhibitors, mucosal protectants, and supportive care, with endoscopic surveillance and dose interruption reserved for higher-grade or refractory injury. Monitoring for the deep cytopenias and infection risk that accompanied lymphodepletion in the randomised trial (17) should be written into institutional protocols before adoption.

## 5 Breakthrough path: engineering, collaboration, and alternative platforms

### 5.1 Engineering of CAR structure and signalling

Armoured CAR-T (TRUCK): CAR-T cells are engineered to secrete immunostimulatory molecules that actively remodel the microenvironment (82): IL-12 recruits and activates innate immunity, reverses the suppressive phenotype, and can eliminate systemic tumours even in the absence of lymphodepletion (97, 98, 117); IL-18 armouring enhances CAR-T persistence and expansion (99, 102); IL-15 supports CAR-T survival and memory differentiation, and membrane-bound IL-15 can maintain a stem-cell-like memory subset via STAT5 independently of CAR signalling (100); secreted checkpoint-blocking fragments can locally relieve suppression within the tumour (136, 137). The challenge lies in balancing efficacy and systemic toxicity, thus most designs employ antigen-inducible local expression.

Signal tuning and suppression of tonic signalling (mechanistic basis detailed in Section 4.4.4): the 1XX ITAM-dose-tuning design (78), TRAC-locus knock-in (80), and inducible degradable/switchable CAR designs to actively create “rest” (73). For the Claudin18.2 target, the instructive example is AZD6422, which incorporated a TGF-β dominant-negative receptor to counter the TGF-β-rich digestive tract microenvironment and showed enhanced persistence and cytolytic function with limited gastric toxicity across several patient-derived xenograft models (103)—on paper a direct answer to the identification of TGF-β as a primary inhibitory axis (Section 4.3.3). The programme is no longer active. AZD6422 entered a phase 1 study in CLDN18.2-positive gastrointestinal tumours, and in July 2025, AstraZeneca discontinued it on efficacy grounds, along with the GPC3-directed AZD5851 (140). No clinical dataset from that trial has been published, so the reason for the shortfall cannot be assigned with any confidence. The outcome is nonetheless consistent with the argument of Section 4.4.3: TGF-β armouring acts on one node of the proposed chain, and relieving one node leaves the rest—antigen heterogeneity, hypoxia, tonic signalling, the NFAT-TOX/NR4A program—untouched. We therefore cite AZD6422 as preclinical proof of principle for TGF-β armouring, and as a caution against expecting any single armouring module to translate, rather than as a clinical-stage product. The common logic of these signal-tuning strategies is to reduce rather than increase signalling—counter-intuitive, yet supported by the most rigorous mechanistic and preclinical evidence (77, 78).

### 5.2 Transcription factor engineering: directly rewriting the exhaustion program

Since exhaustion is driven by a defined network of transcription factors (Section 4.4.1), directly rewriting this network represents the most targeted strategy. Beyond NR4A (67, 72) and TOX/TOX2 (70) knockout, another approach is overexpression of antagonistic transcription factors: the imbalance of the AP-1/bZIP family is a critical node in exhaustion, and overexpression of c-Jun in CAR-T cells confers exhaustion resistance and restores expansion and effector function (84). Transcription factor engineering complements signal tuning (Section 5.1)—the latter attenuates upstream driving signals, whereas the former rewrites downstream programs.

### 5.3 Logic gating and gene editing

Logic-gated CARs fundamentally narrow the scope of killing. “AND-gate” requires simultaneous recognition of two antigens for activation, exploiting combinatorial antigen differences between tumour and normal tissues to achieve selectivity; “NOT-gate” (e.g., the Tmod system) suppresses activation upon recognition of an antigen expressed solely on normal tissues, a design that has been validated for CEA in tumours with allelic loss of the corresponding HLA (86, 90). Synthetic receptors such as synNotch and SUPRA further decouple antigen recognition from effector output, enabling programmable Boolean logic; tuning antigen sensitivity and combinatorial logic gating are considered key engineering directions for simultaneously overcoming heterogeneity and off-tumour toxicity (91, 105, 107). For targets lacking a natural therapeutic window (i.e., not satisfying the three criteria in Section 4.2), logic gating offers a means to artificially create one.

Gene editing provides precision via a “subtractive” approach. In addition to the aforementioned NR4A/TOX knockouts, three microenvironment-related targets are of particular value: knockout of PDCD1 (or disruption via CRISPR, TALEN, etc.) relieves checkpoint inhibition and enhances antitumour efficacy of human CAR-T cells (108, 109); knockout of TGFBR2 renders CAR-T cells “deaf” to TGF-β—given the primacy of TGF-β in gastrointestinal tumours as detailed in Section 4.3.3, this modification is especially well-justified in this tumour group (103), although the justification remains preclinical and the discontinuation of AZD6422 (Section 5.1) is a reminder that it has yet to be borne out in patients (140); knockout of ADORA2A (the A2A receptor) relieves adenosine-mediated suppression, with efficacy superior to shRNA knockdown or pharmacological blockade, and A2AR-deficient CAR-T cells are well-tolerated in mice without overt pathology (92). Given that the strength of adenosine-mediated suppression is comparable with that of TGF-β (Section 4.3.3), A2AR should be ranked alongside PDCD1 and TGFBR2 as a core gene-editing target, rather than an optional supplement. Multiplex editing (knockout of endogenous TCR and HLA, knock-in of CAR) forms the technical basis for universal (allogeneic) CAR-T; the first-in-human study confirmed that CRISPR-engineered T cells with simultaneous knockout of TRAC/TRBC/PDCD1 are safe and can be durably engrafted (112).

Homing engineering: To address insufficient infiltration, co-expression of chemokine receptors (CXCR2, CCR2, CCR4, etc.) matching the tumour chemokine profile can guide CAR-T cells to migrate directionally to the tumour bed (114–116, 118). Incorporating cytokine receptor signalling domains (e.g., STAT-recruiting motifs from IL-2Rβ) provides a third signal and reduces dependence on exogenous cytokines (101).

### 5.4 Combination therapies

Combination with checkpoint inhibitors: PD-1/PD-L1 blockade relieves the inhibitory axes upregulated on CAR-T cells under chronic stimulation. Timing is nevertheless critical, as Section 4.4.2 sets out. Once exhaustion enters a “fixed” state, checkpoint blockade restores function far less effectively (69, 71, 79), so early intervention outperforms salvage in later lines.

Combination with oncolytic viruses: Oncolytic viruses lyse tumours, release antigens, and induce immunogenic cell death. Engineering them to express chemokines or cytokines intratumorally further “heats” cold tumours. Oncolytic adenoviruses armed with RANTES/IL-15 recruit CAR-T cells, support their intratumoural survival, and prolong survival in tumour-bearing hosts (104, 119). In pancreatic ductal adenocarcinoma, mesothelin-directed CAR-T combined with a cytokine-armed oncolytic adenovirus likewise shows synergy (120). This combination is particularly relevant for MSS colorectal cancer and pancreatic cancer.

Combination with chemotherapy/targeted therapy: Moderate cytoreduction reduces antigen load and alleviates exhaustion driven by excessive stimulation (acting upstream of the causal chain in Section 4.4.3), whereas lymphodepletion creates cytokine space for CAR-T expansion.

Improving intratumoural hypoxia: Based on the mechanism outlined in Section 4.4.3, reducing intratumoural hypoxia or alleviating endogenous ROS in T cells could theoretically attenuate the exhaustion drive from the metabolic side and synergise with immunotherapy (76). Three routes are available, and they act at different points of the chain. The first is to raise oxygen delivery. Anti-angiogenic therapy given at low dose prunes the aberrant vasculature described in Section 4.5 and transiently restores perfusion, and the combination of atezolizumab with bevacizumab in hepatocellular carcinoma (8) establishes that this can be done safely alongside immunotherapy; in pancreatic ductal adenocarcinoma, where hypoxia is driven largely by stromal compression of vessels rather than by vascular immaturity, the equivalent manoeuvre is stromal remodelling (135), which is why the infiltration-directed and hypoxia-directed strategies converge in that tumour. The second is to lower oxygen and glucose consumption by the tumour itself. Because the hostile milieu is generated by tumour glycolysis, and because high glycolytic flux marks resistance to adoptive cell therapy (48), suppressing lactate production at source relieves acidification and glucose competition simultaneously and, unlike simply feeding the infused cells, does not also fuel regulatory T cells and M2-polarised macrophages (Section 4.3.1) (47). The third is to protect the CAR-T cell downstream of the insult, by sustaining mitochondrial biogenesis or limiting reactive oxygen species accumulation, since it is mitochondrial failure and the resulting ROS burst, not hypoxia as such, that amplifies NFAT activity (76, 83). Enforced PGC-1alpha expression and antioxidant support have restored function in tumour-infiltrating T cells in preclinical work, and either could in principle be delivered as an armouring module rather than as a systemic agent. Two practical points follow. Sequence matters: because the chain is driven by conditions present at the moment of antigen encounter, perfusion should be improved before rather than after infusion, which argues for a short vascular-normalisation window preceding lymphodepletion instead of concurrent administration. Moreover, stratification matters: intratumoural hypoxia is measurable, by imaging or by a hypoxia gene signature on the diagnostic biopsy, so it can serve both to select patients for a hypoxia-directed combination and as a pharmacodynamic readout of whether the intervention worked (Section 6.1). We note plainly that no CAR-T combination trial in gastrointestinal cancer has yet tested any of this prospectively; node 4 of **Figure 4** remains the one node of the causal chain without a clinical-stage strategy, and on the analysis presented here, that gap is the more consequential omission rather than a minor one.

**Figure 4:**
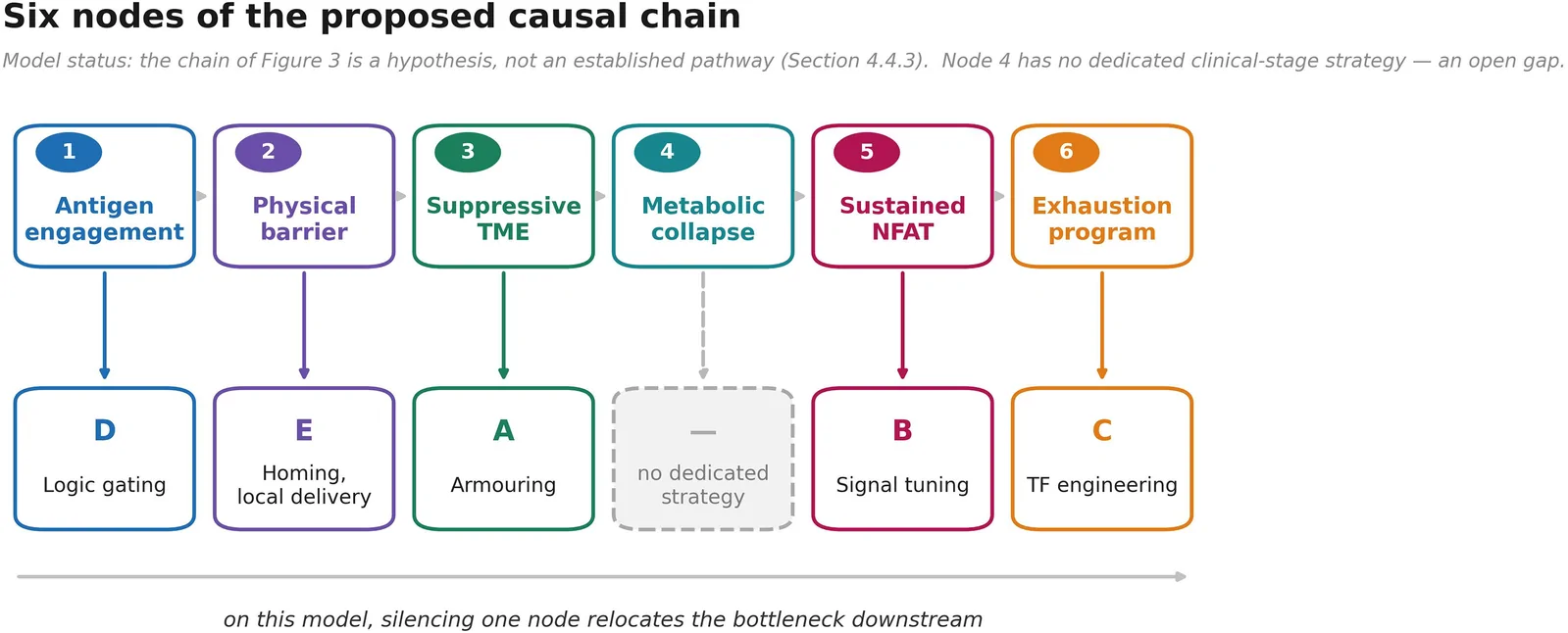
Engineering and combination strategies mapped onto the nodes of the proposed NFAT causal chain. The proposed chain of **Figure 2** resolved into six nodes, each paired with the strategy that acts on it: D, logic gating; E, homing and local or regional delivery; A, armouring; B, signal tuning; C, transcription-factor engineering. Node 4 (metabolic collapse) has no dedicated clinical-stage strategy. Because the nodes lie on one chain, silencing any single node relocates the bottleneck downstream.

Locoregional administration: Regional delivery raises local CAR-T concentrations while reducing systemic exposure. Preclinical work shows that locoregionally delivered mesothelin-directed CAR-T generates potent and durable CD4-dependent antitumour immunity (121). A phase I human study combining intrapleural administration with pembrolizumab confirmed safety and antitumour activity (122). For gastrointestinal tumours, intraperitoneal administration directly addresses peritoneal metastases and clears minimal residual disease after cytoreductive surgery.

Gut microbiome modulation: The microbiota influences immunotherapy efficacy by modulating systemic and local immune status. Rational interventions—probiotics, faecal microbiota transplantation, and avoidance of unnecessary broad-spectrum antibiotics—may therefore enhance CAR-T expansion and function (106, 123). More direct evidence comes from adoptive cell therapy itself. Modulation of the gut microbiota enhances CAR-T antitumour efficacy via CD8α^+^ dendritic cells, IL-12 and antigen cross-presentation, and patients receiving oral vancomycin reached higher peak CAR-T expansion (124, 125). This point is particularly pertinent in gastrointestinal tumours, where the tumour site coincides with the microbiota niche. These combinations are not interchangeable across tumour types (**Figure 5**); matching them to the dominant bottleneck is what the target-tumour matrix (Section 3) implies in practice. Pancreatic ductal adenocarcinoma is dominated by dense desmoplastic stroma and severe hypoxia (Sections 4.5, 4.4.3). The priority there is stroma- and hypoxia-directed: CAR-macrophages or CXCR2-armed CAR-T for infiltration, oncolytic viruses to remodel the stroma, and local or intraperitoneal delivery. Checkpoint blockade ranks lower, because these tumours remain largely cold. In microsatellite-stable colorectal cancer, TGF-β-activated stroma and T-cell exclusion form the primary evasion mechanism (Section 4.3.3). TGF-β-directed engineering—TGFBR2 disruption or a dominant-negative receptor—paired with an oncolytic or other heating agent is therefore the more rational combination, and checkpoint blockade is best reserved for the dMMR/MSI-H subset. In gastric and gastro-oesophageal cancer, satri-cel already has single-agent activity. The highest-yield additions there pre-empt exhaustion: checkpoint blockade given early rather than as late salvage (Section 4.4.2), moderate cytoreductive bridging, and above all earlier-line positioning (Section 6.3). **Table 3** summarises how the alternative platforms differ on the practical axes—persistence, cost, manufacturing complexity, and regulatory pathway—that also bear on these choices.

**Figure 5:**
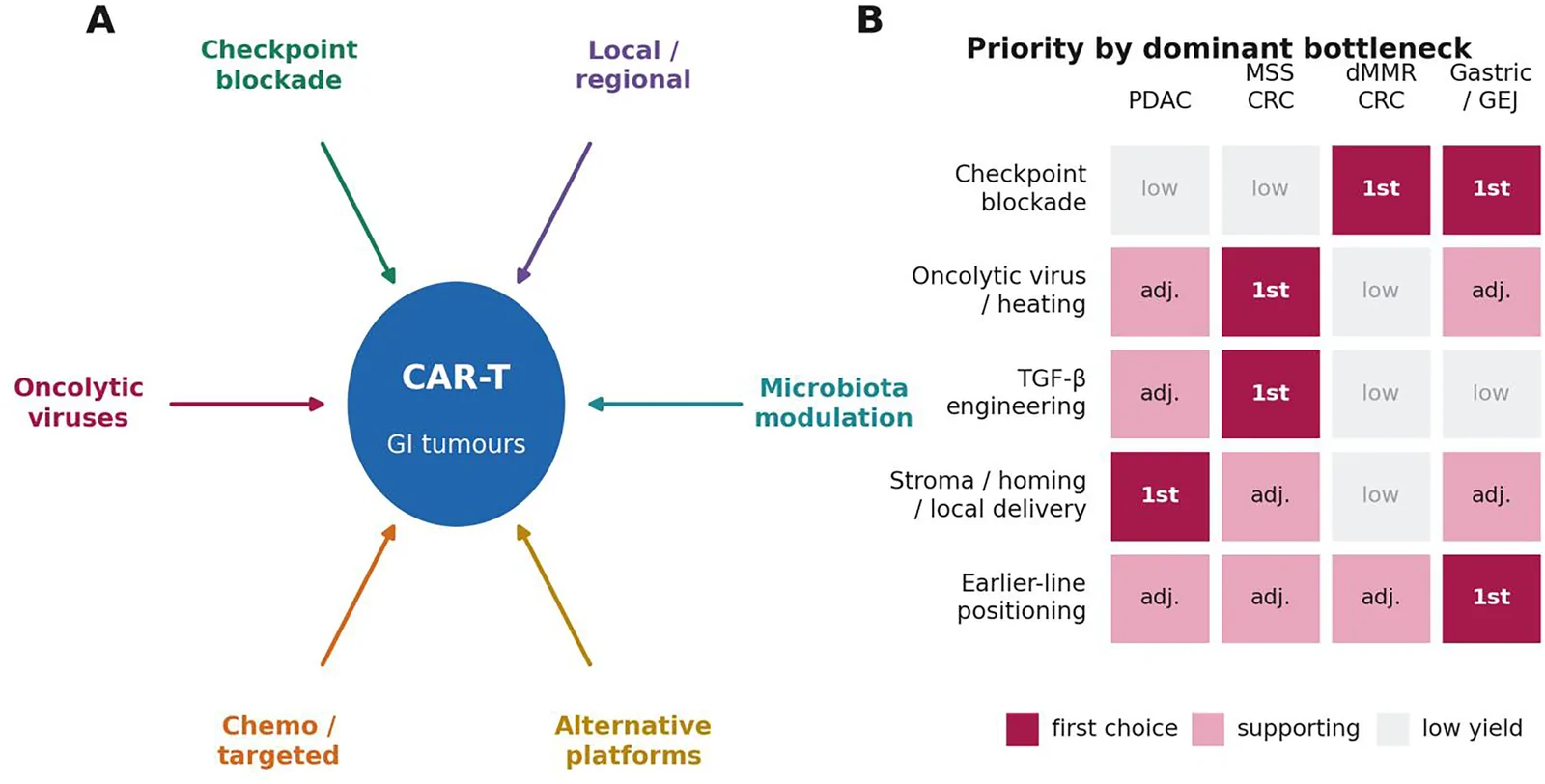
**(A)** Combination partners and alternative platforms for CAR-T in gastrointestinal tumours. **(B)** Priority of each approach by the dominant bottleneck of the tumour type. PDAC, pancreatic ductal adenocarcinoma; CRC, colorectal cancer; MSS, microsatellite stable; dMMR, mismatch-repair deficient; GEJ, gastro-oesophageal junction.

**Table 3:** CAR-T and alternative cellular platforms compared across persistence, cost/manufacturing, and regulatory pathway.

Platform	Persistence	Cost and manufacturing	Regulatory pathway	Best-fit role (GI)	Ref
Autologous CAR-T (satri-cel)	Months; capped by exhaustion	High; individualised, long vein-to-vein	Approved (NMPA 2026)	Reference platform; late-line, moving earlier	(16–18)
Allogeneic (universal) CAR-T	Often shorter unless edited	Off-the-shelf; multiplex editing adds complexity	Investigational; extra gene-editing review	Access at scale; bridging-failure mitigation	(112, 134)
CAR-NK	Short; IL-15/CISH editing extends	Off-the-shelf; lower CRS	Investigational; lower-toxicity profile	Lower-toxicity option; complements CAR-T	(111, 127, 129)
CAR-macrophage (CAR-M)	Non-proliferative, single-wave	Autologous myeloid; no long expansion	Early investigational	Stroma-rich tumours (PDAC); infiltration + presentation	(110)
*In vivo* CAR-T (mRNA-LNP)	Transient by design	Potentially lowest; no ex vivo culture	Early; drug-like model	Population-scale access; “younger” cells	(113, 131)

The platforms are complementary rather than mutually exclusive; the choice depends on the dominant bottleneck (Section 5.4) and on access constraints (Section 6.2). Primary references for each platform are listed in the final column.

### 5.5 Alternative cellular platforms

CAR-macrophages (CAR-M) retain the innate tissue-infiltrating, phagocytic, and antigen-presenting capacities of macrophages. In densely stromal tumours (particularly pancreatic cancer; see Section 4.5), their infiltration potential surpasses that of T cells, and they can remodel the myeloid microenvironment and elicit endogenous T-cell responses through antigen presentation (110). CAR-NK cells possess MHC-independent cytolytic capacity; when derived from allogeneic sources, they carry a low risk of graft-versus-host disease, are amenable to “off-the-shelf” manufacturing, and exhibit a relatively lower CRS risk; co-expression of IL-15 coupled with knockout of its negative feedback checkpoint CISH can further enhance their metabolic fitness and antitumour activity (111, 127, 129). These platforms are complementary to, rather than replacements for, CAR-T.

### 5.6 *In vivo* CAR-T generation: a paradigm shift in accessibility

Platforms such as mRNA-lipid nanoparticles (mRNA-LNP) enable direct, transient generation of CAR-T cells *in vivo*, bypassing the entire ex vivo process of collection, transduction, and expansion (113). If matured, this approach would fundamentally alter the cost structure, manufacturing turnaround time and accessibility of CAR-T therapy. An additional, less frequently mentioned theoretical advantage is that transient *in vivo* generation circumvents the tonic signalling and pre-exhaustion accumulated during prolonged ex vivo culture (Section 4.4.4), potentially yielding “younger” cells for infusion (131). For gastrointestinal tumours, with patient populations numbering in the millions, this may carry greater population-level significance than any single efficacy improvement.

## 6 Translation and accessibility

### 6.1 Efficacy biomarkers: selecting the right patients for CAR-T

Following regulatory approval, the central question in the real-world setting becomes “who will benefit” (128). Candidate predictive factors include: target antigen density and homogeneity (rather than a binary “positive” classification); baseline tumour burden; the proportion of memory-like/stem-cell-like T cells within the infused product, as well as its degree of pre-exhaustion—in haematological malignancies, enrichment of early-memory T cells in the infusion product correlates with complete response, and product characteristics track with both efficacy and toxicity (132, 133) (the latter can be quantified using the transcriptional/epigenetic markers described in Section 4.4 (61, 62, 74)); immune infiltration and hypoxia features of the microenvironment; and host factors such as recent antibiotic exposure affecting the microbiota. Standardisation of companion diagnostics—particularly the discrepancy in immunohistochemical cutoffs noted in Section 3.1—is a prerequisite for all of these.

### 6.2 Manufacturing, cost, and accessibility

The individualised manufacturing process for autologous CAR-T is lengthy and costly, and its globalisation and standardisation constitute a systemic engineering challenge (134), posing a genuine accessibility bottleneck in gastrointestinal tumours with their vast patient populations. Universal (allogeneic) CAR-T, CAR-NK, and *in vivo* CAR-T generation represents three parallel pathways to overcome this bottleneck. In addition, disease progression during the manufacturing waiting period (“bridging failure”) is a common issue in clinical practice, requiring concurrent optimisation of manufacturing speed and bridging regimens. Bridging therapy during the manufacturing window should control disease without exhausting the T-cell compartment or expanding antigen-loss clones: moderate-intensity chemotherapy or targeted agents are preferable to aggressive regimens, consistent with the argument (Section 5.4) that cytoreduction lowers antigen load and eases exhaustion pressure. On cost-effectiveness, autologous CAR-T that shifts median progression-free survival from 1.77 to 3.25 months in a heavily pretreated population—a 1.48-month difference between medians, hazard ratio 0.37 (95% CI 0.24-0.56)—sits at the margin of conventional value thresholds; the case strengthens materially only if the earlier-line and adjuvant settings (Section 6.3) convert that benefit into durable disease control, or if allogeneic, CAR-NK, and *in vivo* platforms lower per-patient manufacturing cost. A formal analysis is indication- and health-system-specific and beyond the scope of this review, but the direction is clear: at current cost and current benefit, patient selection (Section 6.1) and line-of-therapy positioning are what determine value.

### 6.3 Advancing to earlier lines: a mechanistically grounded strategy

As noted in Section 2.3, CAR-T is moving from late-line salvage toward adjuvant, consolidation and first-line sequential settings. The analysis in Section 4 provides a mechanistic rationale for this strategy, beyond empirical observation: lower tumour burden translates into lower antigen stimulation intensity, and smaller tumours entail milder hypoxia—precisely the two conditions required to drive exhaustion are simultaneously attenuated (Section 4.4.3); concurrently, patients harbour better T-cell reserves, exhaustion remains in a plastic phase, and interventions such as checkpoint blockade remain effective (Section 4.4.2). If the early signals from adjuvant therapy (21) are confirmed in larger cohorts, the value of CAR-T in gastrointestinal tumours will be elevated from “prolonging survival in the advanced setting” to “increasing cure rates”—two qualitatively different objectives.

## 7 Conclusion

In June 2026, the approval of satri-cel closed a debate that had persisted for over a decade: CAR-T can work in solid tumours, provided the right target is chosen. That Claudin18.2 became the “right target” was not coincidental—its epitope is structurally masked by tight junctions in normal tissues, its expression spares the gastric mucosal stem cell zone, and its host tissue is regenerable and monitorable. These three criteria constitute a generalisable target-screening paradigm, whose value may extend beyond this single product.

Yet equally sobering are the numbers: median progression-free survival of 3.25 months (95% CI 2.86-4.53) versus 1.77 months (95% CI 1.61-2.04), a difference between medians of 1.48 months, with a hazard ratio of 0.37 (95% CI 0.24-0.56). The watershed has been crossed, but substantial obstacles remain. This review proposes that the efficacy ceiling is not simply the sum of four isolated obstacles. In the model we advance, persistent antigen stimulation and intratumoural hypoxia converge at the molecular level on NFAT and jointly drive the TOX/NR4A-dominated exhaustion program; stromal barriers block infiltration and worsen hypoxia, feeding the same program; and antigen heterogeneity allows escape clones to survive selective pressure. On this reading, the four constraints are mutually reinforcing rather than parallel. We restate that the chain has been assembled from chronic infection and mouse tumour systems, that it has not been tested in human CAR-T cells or in satri-cel-treated patients, and that it should be read as a framework to be tested—by measuring mitochondrial function, ROS, and NFAT-target gene expression in CAR-T cells recovered from treated patients—rather than as an established explanation.

The practical implication, if the model holds, is that single-point interventions will be of limited effect. True breakthroughs require simultaneous attenuation of upstream drivers (reducing tonic signalling, tuning signal intensity, and alleviating antigen load and hypoxia), rewriting of downstream programs (TOX/NR4A knockout and c-Jun overexpression), broadening of the therapeutic window (logic gating), and improvement of access and persistence (homing engineering, armouring, and alternative platforms). Advancing CAR-T to earlier lines is the most promising current strategy precisely because it acts on several nodes of the proposed chain at once.
